# Progress of genome-wide association studies of ankylosing spondylitis

**DOI:** 10.1038/cti.2017.49

**Published:** 2017-12-01

**Authors:** Zhixiu Li, Matthew A Brown

**Affiliations:** 1 Translational Genomics Group, Institute of Health and Biomedical Innovation, School of Biomedical Sciences, Queensland University of Technology at Translational Research Institute, Brisbane, Queensland, Australia

## Abstract

Ankylosing spondylitis (AS) is an immune-mediated arthritis which primarily affects the spine and sacroiliac joints. Significant progress has been made in discovery of genetic associations with AS by genome-wide association studies (GWAS) over past decade. These findings have uncovered novel pathways involved pathogenesis of the disease and have led to introduction of novel therapeutic treatments for AS. In this Review, we discuss the genetic variations associated with AS identified by GWAS, the major pathways revealed by these AS-associated variations and critical cell types involved in AS development.

## Introduction

Ankylosing spondylitis (AS) is a common, chronic, highly heritable, immune-mediated arthritis that affects primarily the spine and sacroiliac joints. It can also affect peripheral joints and extra-articular tissues including the eye, gut and skin. AS is the prototypic form of spondyloarthritis (SpA), a group of rheumatic disorders which share clinical, genetic and radiographic features, and which includes psoriatic arthritis (PsA), reactive arthritis (ReA), arthritis of inflammatory bowel disease (IBD), juvenile-onset spondyloarthritis and undifferentiated Spondyloarthritis (USpA). The diagnosis of AS is determined by clinical and radiographic features as defined by the modified New York (mNYAS) classification criteria.^1^ This review focuses on mNYAS criteria based studies because multiple lines of evidence demonstrate that classification criteria recently designed to increase sensitivity in early disease^2^ lack adequate specificity^3, 4^ and lead to marked increases in genetic heterogeneity.^5^

The prevalence of AS varies in different countries, being highly correlated with the frequency of the class I major histocompatibility complex (MHC) allele human leucocyte antigen (HLA)-B*27.^6^ The prevalence of AS in European-descent populations is about 0.55%,^7^ 0.2–0.54% in Han Chinese populations^8, 9^ and 0.074% in South Africa.^10^ AS affects men more often than women and the ratio of men with AS to women is about 2–3:1,^11, 12, 13^ while the ratio in Europe is 3.8:1 and in Asia is 2.3:1.^10^ In addition, men are generally more likely to have a younger onset age and more often develop extra-articular features such as IBD and psoriasis.

## Genetic epidemiology of ankylosing spondylitis

AS has been known to run strongly within families, for a long time. The risk ratio of the sibling or first-degree relative of an AS patient is >52 compared with the general population (non-related individuals) risk.^14, 15, 16^ It was unclear whether the co-familiality was due to shared environment or genetic factors until the association of HLA-B*27 allele with AS was discovered in the early 1970s.^17, 18, 19^ The recurrence risk drops rapidly with increasing distance of relationship to the proband (monozygotic (MZ) twins 63%, first-degree relatives 8.2%, second-degree relatives 1.0% and third-degree relatives 0.7% in Europeans;^14^ first-degree relatives 3.84%, 2nd degree relatives 0.87% and 3rd degree relatives 0.315% in Han Chinese).^20^ The evidence suggests that AS is a polygenic rather than monogenic disease since the frequency of monogenic disease reduces about half with each increase in distance of relationship to the proband, while frequency in polygenetic disease reduces approximate the square root with each increase in distance of relationship to the proband. Also, the concordance rate in dizygotic twins (DZ, 12.5%), or even in HLA-B*27 positive DZ twins (24–27%), is much lower than in MZ twins (63%), implying the presence of non-HLA*B27 factors, either environmental factors or other non-HLA-B*27 genes influencing disease susceptibility.^14, 21^

The estimated heritability of AS by twin studies is >90%.^21^ The variants associated with AS from that study explain 27.82% of AS heritability, with the greatest contribution coming from the MHC (20.44%) and with 7.38% coming from non-MHC loci.^22, 23^ Disease activity (BASDAI, 51%),^24^ functional impairment (BASFI, 68%),^24^ radiographic change (62%),^25^ and age of symptom onset,^26^ all additionally show significant heritability in AS.

The co-existence of AS and IBD has been known for a long time.^27^ Clinically diagnosed IBD presents in 5–10% of the AS patients, and 40–60% of AS patients have developed subclinical inflammation in gut and bowel.^28^ Moreover, the risk ratios of IBD were 3.0 and 2.1 in first- and second-degree relatives of patients with AS compared with unrelated individuals, respectively.^15^ These findings suggest that these two diseases may have similar aetiology, and multiple genes shared by these two diseases have been found.^22, 29^ Studying the heritability captured by the Immunochip SNP microarray, strong co-heritability was observed between AS and Crohn’s disease (40% including and 39% excluding the MHC), ulcerative colitis (33 and 31%) and to a lesser but nonetheless significant extent with psoriasis (27 and 20%) and primary sclerosing cholangitis (23 and 20%).^22^

## HLA-B*27

The association of AS with HLA-B*27 was discovered in the early 1970s^17, 18^ and it is one of the strongest genetic associations with any common human disease. The prevalence of HLA-B*27 varies in different ethnic groups and populations. The population prevalence of HLA-B*27 is approximately 8% in British,^30^ 4% in black Africans,^31^ and 3.6–5.7% of Han Chinese.^8^ In general, the population prevalence of AS parallels the frequency of HLA-B*27 except in West Africans.^32^ 80-95% of AS patients of European ancestry are HLA-B*27 positive.^33^ Despite the strong association between HLA-B*27 and AS, only 2–5% of HLA-B*27 positive individuals develop AS, suggesting that other factors such as other loci, environmental or stochastic factors also contribute considerably to AS development.^6, 34^ *HLA‐B27* homozygosity moderately increases risk of AS compared with *HLA‐B27* heterozygosity.^23, 35, 36^

The introduction of high-throughput *HLA* sequencing has revealed that HLA-B*27 is remarkably polymorphic. To date, at least 271 HLA-B*27 subtypes (B*27:01-B27:164) have been reported^37^ and the number keeps increasing rapidly. Most are too rare to determine whether they are AS-associated or not, but two subtypes have been shown to have neutral associations with AS, HLA-B*27:06 (a common subtype in south-east Asia),^38^ and HLA-B*27:09 (a rare subtype found primarily on Sardinia).^39^ HLA-B*27:05 is present in nearly all populations^40^ and it is suggested to be the possible ancestral allele. HLA-B*27:05 and HLA-B*27:02 are the main subtypes associated with AS in Caucasians, in Asians the main associated subtypes are HLA-B*27:04 and HLA-B*27:07 and in Mediterranean populations is HLA-B*27:02.^41^ HLA-B*27 positive patients tend to develop AS earlier than HLA-B*27-negative patients.^35, 42^

## Non-HLA-B*27 MHC associations

There are clearly other HLA alleles, and potentially MHC genes, associated with AS. HLA-B*60 was the first non- HLA-B*27 alleles identified to be associated with AS in HLA-B*27 positive patients,^43^ and was later confirmed in HLA-B*27 positive UK cases.^44^ In addition to HLA-B*60, HLA-B*61 was identified to be associated with HLA-B*27-negative Taiwan Chinese AS patients.^45^ Association of HLA-B*39 with HLA-B*27 negative AS patients in Japanese was observed^46^ in a small cohort, but was not replicated in European-descent cases.^44^

Though the associations of these *HLA-B* alleles with AS were identified, the complexity of MHC region makes it is extremely difficult to further study the associations between MHC loci and diseases until new technology and large study cohort are available. Immunochip is an Illumina Infinium SNP microarray with 195 806 SNPs and 718 small insertion–deletions on the array. The chip has dense coverage of the MHC and killer immunoglobulin-like receptor (*KIR*) loci.^47^ Dissection of the associations of MHC with diseases has been greatly improved with the help of Immunochip. Other than these *HLA-B* alleles, HLA-B*51:01, HLA-B*47:01, HLA-B*40:02, HLA-B*13:02 and HLA-B*40:01 were identified to be associated with increasing risk of AS whilst HLA-B*07:02 and HLA-B*57:01 were found to be associated with reduced risk of AS in a study cohort with 9,069 AS cases and 13,578 population controls of European descent using Immunochip.^37^

Furthermore, non-HLA*B alleles, *HLA-A* (HLA-A*02:01), *HLA-DPB1* and *HLA-DRB1* were associated with AS after controlling for the associated *HLA-B* haplotypes in this study.^37^ HLA-B51 is also a known risk factor for the spondyloarthritis-related disease, Behcet’s disease, suggesting some shared pathogenic mechanisms with AS. HLA-B*7 has been shown to be highly protective against AS in Chinese (odds ratio after control for HLA-B*27=0.15).^48^ Another study in the Korean population using Immunochip reported that HLA-C*15:02 allele was associated with AS in addition to HLA-B*27.^49^

## Non-MHC genetic associations

Enabled by the development of high-throughput chip-based genotyping and computational genetics from early 2000s, genetic studies were able 10 years to ago to move to a new era, from family linkage study to genome-wide association studies (GWAS). In this review we consider not only association studies using the GWAS chip but also on customized chips, such as the Illumina Immunochip.^47^ Table 1 lists the published key AS GWAS/association studies since 2007. The first AS GWAS was conducted by the Wellcome Trust Case Control Consortium (WTCCC) and Australo-Anglo-American Spondylitis Consortium (TASC) in 2007, analysing 14 500 non-synonymous SNPs in 1,000 AS patients and 1500 healthy controls. This study made the first definitive identification of non-MHC susceptibility loci in AS, these being SNPs in endoplasmic reticulum aminopeptidase 1 (*ERAP1*) and interleukin 23 receptor (*IL23R*).^50^

A subsequent and comprehensive GWAS on a larger cohort of 2,053 AS patients and 5,140 controls of European ancestry was performed by TASC in 2010.^33^ This study replicated the previous findings of *ERAP1* and *IL23R*. Moreover, additional loci, including Anthrax Toxin Receptor 2 (*ANTXR2*), Interleukin 1 Receptor Type 2 (*IL1R2*) and two intergenic regions on *2p15* and *21q22* were also found to be associated with AS at genome-wide significance. The association with intergenic regions suggests that they may harbour non-coding functional elements or be involved in regulating the expression of other genes. Further studies are needed to investigate their roles in AS pathogenesis.

In 2011, TASC and WCCC2 published the largest AS GWAS to date, involving a cohort of 3,023 AS patients and 9,141 health controls of European descent.^51^ This study identified several new associations, with its larger cohort. For the first time, *RUNX3, LTBR-TNFRSF1A* and *IL12B* were reported to be associated with AS at genome-wide significance level. It also replicated the HLA-B*27, *ERAP1, IL23R, 2p15, 21q22* and *KIF21B* findings.

In 2013, International Genetics of Ankylosing Spondylitis Consortium (IGAS) conducted a case–control AS association study on Immunochip which has dense coverage in immune loci and MHC region. This cohort contains 10 619 AS cases and 15 145 controls in populations of European, East Asian and Latin American ancestry.^23^ IGAS identified 13 new associations of AS with replication of 11 previous findings other than HLA-B*27. These new associations are with SNPs nearby or within *IL6R, FCGR2A, UBE2E3, GPR35, BACH2, ZMIZ1, NKX2-3, SH2B3, GPR65, SULT1A1, NOS2, TYK2* and *ICOSLG*.

In 2016 an AS association study using the Illumina Exomechip was conducted in a cohort of 5,040 AS patients and 21 133 healthy controls of European descent.^52^ Technically, this is not a genome-wide study but an exome-wide study. Exomechip has dense coverage of low-frequency and rare variants in addition to common coding variants. Due to the limited power of detecting rare variants and incomplete coverage of rare variants, this study only identified two novel loci (*USP8* and *CDKAL1*) associated with AS at genome-wide significance and confirmed 11 known AS associations.^52^

**Figure 1:**
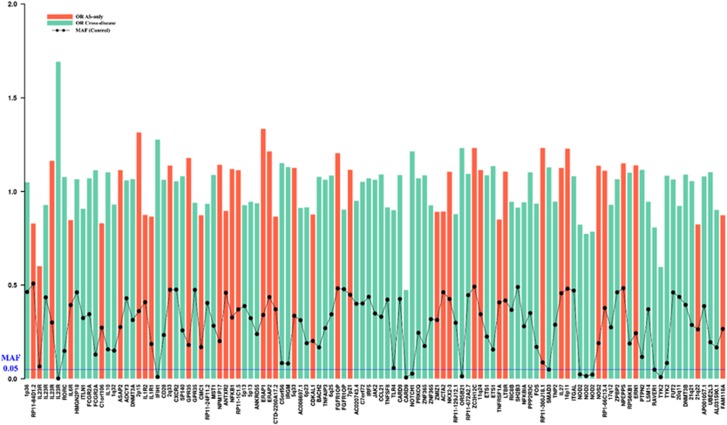
Odds ratios and minor allele frequencies in controls of 113 non-MHC AS-associated variants identified in Ellinghaus *et al.*^22^ Each bar and dot represents a GWS variant. The variants are labelled by nearby genes. The red and green bars respectively represent odds ratio of AS-associated variants from AS-only case–control analyses, and analyses leveraging pleiotropy. Black dot represents minor allele frequency in healthy controls. The blue line indicates 5% minor allele frequency.

More recently, the largest AS case–control association study on Immunochip identified 113 AS-associated genome-wide significant variants by combining case cohorts from five related diseases (Crohn’s disease, psoriasis, primary sclerosing cholangitis and ulcerative colitis).^22^ This study has 8,726 AS cases and 34 213 health controls, and another 43 536 cases in other four diseases on Immunochip. 39 out of these variants are GWS significant in AS-only cohort, while additional 74 non-MHC GWS variants were identified in pleiotropy analysis with other four diseases then confirmed as being independently AS-associated. The larger sample size of this study increased its power to detect loci in disease-specific analyse, and to an even greater extent in analyses of pleiotropic genetic effects. It identified 17 new loci in AS missed in the original Immunochip study simply through the use of a much larger number of controls. The new AS-associated loci are *ITLN1, CTLA4, CMC1, NPM1P17, NFKB1, CDKAL1, FGFR10P, 6p22, 7p21, ACTA2, 11q24, PPP2R3C, CORO1A, 16p11, ERN1, PTPN2* and *FAM118A* (for which suggestive association had already been identified in the AS Exomechip study). Odds ratios and minor allele frequencies in controls of 113 non-MHC variants identified in this study are illustrated in Figure 1. Given its large size, this study was sufficiently powered to identify several rare variants, including four variants with MAF < 1% and seven with MAF 1–5%. Notably, all the rare variants were identified by pleiotropic analysis with multiple diseases.

Thus far, most AS GWAS or case–control association studies have been performed in white European cohorts. The only GWAS yet carried out in the Han Chinese population, reported in 2011, identified two novel genome-wide significant non-MHC loci (*2p15*, *5q14.3* and *12q12*), and replicated the known HLA-B*27 and *2p15* loci.^53^ The two novel non-MHC loci were not replicated in the IGAS Immunochip study (either in European descent or a large Chinese case–control analysis), nor the cross-disease study mentioned above, and are thus likely to be false positives.

There is clearly great need for a large GWAS to be performed in Han Chinese population to leverage the difference in genetic makeup of east-Asians to identify further genes and pinpoint AS-genetic associations. For example, it has been shown that the *IL23R* SNPs associated with AS in European descent populations are not associated with AS in Han Chinese.^54^ Subsequent studies have shown that this is likely because the key European-associated variant, rs11209026, is not polymorphic in east Asians. Rather, a different variant (rs76418789, G149R) is AS-associated in east Asians.^55^ This variant shows weak association in European descent populations too but that effect is difficult to see because of the strength of association of other variants at this locus, notably rs11209026 (R381Q).

There is significant gender bias in AS and women are generally more resistant to getting AS than men. In addition, differences in recurrent risk between the offspring of affected mothers and fathers have been observed. These findings imply loci of X-chromosome may contribute to disease susceptibility. Despite multiple AS GWAS having been performed on autosomes, there are only two AS association studies on X chromosome, each in small cohorts. Thus far, no association or linkage of the X-chromosome with AS have been found.^56, 57^ Therefore, the sex bias in AS hasn’t been explained by X-chromosome–encoded genetic effects, although no formal high-density SNP analysis of this chromosome has yet been reported.

Identified AS susceptibility loci are involved in numerous immunomodulatory pathways affecting innate and adaptive immunity, such as antigen presentation and binding, TNF-α/NF-κB activation and signalling, IL-23R signalling, lymphocyte development and activation, IL-1 cluster genes, G-protein coupled receptor and IL-17/IL-22 mediated immunity. Though there are over 100 AS-associated loci that have been identified, the underlying mechanism by which they are involved remain unclear for most of these loci. There are extensive ongoing studies try to pinpoint the causative variants of AS, understand their roles in AS, and develop new therapeutic treatment for AS. Among these pathways, MHC class 1 presentation and IL-23 pathway are major pathways involved in AS development and also the best studied.

## Aminopeptidase in AS

*ERAP1* is one of the three aminopeptidases (other two are *ERAP2* and *NPEPPS*) have been shown to be associated with AS. Association of *ERAP1* with AS was firstly identified by WTCCC in European Caucasian population in 2007.^50^ It was a big leap in AS research since it is one of the two first AS-associated non-MHC genes. This association has been widely replicated different ethnics groups, including (not exclusively) Han Chinese,^53, 54^ Korean,^58^ white European ancestry^59, 60^ and Iranian populations,^61^ suggesting common *ERAP1* variants are involved in AS rather than diverse rare variants. A recent study investigated haplotypes generated from five common *ERAP1* SNPs—rs2287987 (M349V), rs30187 (K528R), rs10050860 (D575N), rs17482078 (R725Q) and rs27044 (Q730E), and further confirmed that the association of *ERAP1* with AS is primarily contributed by individual common *ERAP1* genotypes, rather than by haplotypes.^62^

ERAP1 operates in AS by acting as a molecular ruler, trimming peptides to optimal length (10-16 residues) for presentation by HLA class 1 molecules.^63^ The main *ERAP1* variant rs30187 only shows association with AS when HLA-B*27 or HLA-B*40 is present.^37^ Interaction between *ERAP1* and HLA-B*27 in AS indicates that peptide trimming and presentation contribute to disease susceptibility. Multiple variants in *ERAP1* have been found to influence the risk of AS. The non-synonymous SNP rs30187 (K528R) is directly associated with AS.^51^ An independent association is observed with rs10050860 (D575N), but this is in a very strong LD with a range of other SNPs and it is unclear if itself is directly disease associated.^51^ According to ERAP1 enzyme structure studies, ERAP1 variants that are associated with AS are commonly located in the active site of enzyme, on the inner surface of the C-terminal cavity and junctions.^64^ The SNP rs30187 is located at the junction domain and it could indirectly affect specificity or enzymatic activity by altering the conformational change between open and closed forms.^65^ Indeed, the protective alleles of rs30187 and rs17482078 (R725Q) are reported to be associated with 40% reduction in enzyme activities of peptide trimming *in vitro* using recombinant ERAP1 protein, whereas rs10050860 showed no effect.^51^

These studies show that disease-associated coding variants in *ERAP1* are gain-of-function. The further molecular and immunological mechanisms by which these variants influence AS risk are the subject of active research and have been reviewed elsewhere. It is also clear that *ERAP1* expression is strongly affected by genetic variation at the locus, something first reported by Myriad Genetics^66^ and subsequently confirmed by others.^67, 68^ Thus it is likely that the genetic variants influencing AS risk at this locus do so by a combination of effects on *ERAP1* expression and function.

Apart from *ERAP1*, polymorphisms of *ERAP2* are also found to be associated with AS in multiple studies.^50, 69^ Particularly, the protective allele (G) of rs2248374 affects the exon 10 splicing site and produces a truncated mRNA. This truncated mRNA is degraded leading to a complete absence of ERAP2. Unlike *ERAP1*, *ERAP2* plays a role in both HLA-B27 positive and negative AS.^70^ Though further studies are needed to understand precise mechanism of how *ERAP1* and *ERAP2* affect AS status, this finding together with the protective effects of rs30187 implies that inhibiting these proteins may be potential treatment for AS.

*NPEPPS* encodes puromycin-sensitive aminopeptidase or alanine aminopeptidase, which is expressed in the cytosol. NPEPPS knockdown has been shown to influence cell surface HLA Class I expression, either increasing or decreasing cell surface expression depending on the HLA Class I antigen involved, and indirectly, through studies of HLA Class I complex stability, to influence the peptide pool available for presentation.^71^

## IL-23 pathway

*IL23R* was first reported to have association with AS in 2007 and this finding revealed the involvement of IL-23 pathway in AS pathogenesis.^50^ It encodes the receptor for a proinflammatory cytokine IL-23 which plays important roles in activation of various proinflammatory cells, including T_h_17 T cells, gamma-delta T cells, NK cells, mast cells, Paneth cells and others.

Multiple variants involved with IL-23 pathway genes, such as *TYK2, JAK2, IL12B, IL6R, IL27, PTGER4* and *CARD9* have also been found to be associated with AS.^23, 72, 73^ TYK2 is a member of Janus kinases (JAKs) protein families and it plays important roles in transducing a range of signals, including IL-23, IL-10, IL-6, IFN-α and -β and IL-12 signals.^74^ *JAK2* encodes the component of JAK-STAT signalling pathway, which is the downstream pathway of IL-23R. *IL12B* encodes IL-12p40, one of the subunits of IL-23 heterodimer. CARD9 mediates the downstream signalling of Dectin-1 which occurs in response particularly to fungal stimulation, leading to expression of pro-inflammatory cytokines.^75, 76^ As the receptor for IL-6, IL-6R is involved in the IL-6 signalling pathway which affects T helper (T_h_)17 cell differentiation amongst other things. Activation of Prostaglandin E2 receptor 4 (EP4), the protein product of *PTGER4* promotes development of T_h_17 cells by decreasing IL-12p70 and increases IL-23 expression level.^77^ IL-27 is in the same IL-12 family with IL-23. It has widespread immunological effects, including promotion of T_h_1 differentiation, and inhibition of T_h_2 cell and T_h_17 differentiation.^78^ Its expression is increased in response to LPS interacting with TLR4, which is itself AS-associated, and by prostaglandin E2 (reviewed in Wang *et al.*).^79^

Multiple genes in this pathway show pleiotropic effect in different diseases. For example, *IL23R* is known to be associated with AS-related diseases including IBD^80^ and psoriasis.^81^ Variants in *TYK2* are associated with AS, IBD, psoriasis, type 1 diabetes, multiple sclerosis and rheumatoid arthritis.^82^ Multiple genes related with *IL23R* pathway are shared between IBD and AS, including *IL23R, IL12B, TYK2, JAK2, IL27* and *CARD9,* highlighting that the two diseases probably share similar aetiology through IL-23R pathway.^29^

IL-23 was reported to have higher expression level in the terminal ileum of AS patients and CD patients^83^ and mouse model studies have revealed that overexpression of IL-23 alone can cause spondyloarthritis.^53, 84^ Sherlock *et al* identified a population of IL-23-responsive cells from entheseal expressed IL-17 and IL-22 that developed in response to the IL-23 treatment.^84^ Clinical paw swelling scores were reduced by inhibiting IL-17 or IL-22, especially by inhibiting both. Over-expression of IL-22 caused disease but overexpression of IL-17 did not. Other animal models of AS, both rat and mouse, have also been shown to be significantly affected by IL-23 pathway targeted interventions.^85^ Clinical trials of therapeutic treatments targeting IL-23 pathway shows promising results, with secukinumab being widely used now in treatment of AS, and multiple other agents in development and trial. It will be very interesting to see in humans the differential effect of targeting IL-23, IL-22 and IL-17, which is hard to predict from genetic studies alone.

## Critical cell types involved in AS

Similar to other complex trait associated polymorphisms, the vast majority of AS GWS variants lie within intergenic regions and therefore raise a great challenge to unravel the mechanisms through which they operate. For instance, AS-associated loci *2p15* and *21q22* are located in intergenic regions which makes it is difficult to pinpoint the associated genes, let alone to elucidate the underlying biological function and involved pathways. Nevertheless, recent studies have indicated that disease-associated variants often contribute through effects on gene regulatory regions in specific cell types or tissues rather than directly modifying the protein function, such as regulating gene expression level by expression quantitative trait loci (eQTL) effect or by altering the accessibility of chromatin.^72, 86^ Though the DNA sequence is the same among all the cells and tissues, the regulatory effect of a variant could be different between cell types and tissues. In addition, with the rapidly increasing availability of epigenetic and gene expression data in hundreds of cell types or tissue types, disease-associated variants can be further investigated in the cell types which they primarily operate.

Various cell types have been suggested to be involved in AS, including CD4^+^, CD8^+^ and regulatory T cells,^87^ T_h_17 cell,^88^ natural killer (NK) cells,^89^ gamma-delta cells,^90^ innate lymphoid cells (particularly ILC3 subset),^91^ B cells,^92^ mast cells^93^ and intestinal Paneth cells.^83^ These studies indirectly reveal cell types which AS-associated variants predominantly function. However, understandably they have mainly focused on circulating immune cells and tissues that are easily accessed.

A role for NK and CD8 T-cells has been suggested both by evidence that these KIR3DL2 bearing cells of these types interact with HLA-B27 dimers leading to IL-17 production.^89^ Further, AS is associated with *TBX21* which encodes T-bet, a transcription factor with effects on differentiation and function of multiple immunological cell types. NK and CD8 T-cells expressing T-bet are increased in AS cases, risk variants of *TBX21* lead to increased expression in AS cases, and *TBX21* knockout mouse are resistant to the development of spondyloarthritis and IBD in the Skg mouse model.^94^

To identify the key cell types involved in the mechanism by which AS-associated genes influence its aetiopathogenesis, we recently integrated AS-genetic and cell-type specific data of epigenomes, transcriptomes and proteomes in a wide range of cell types.^95^ This analysis suggested that AS-associated variants primarily operate through effects in CD8^+^ T cells, CD4^+^ T cells, NK cells, regulatory T cells, monocytes, as well as gastrointestinal cells. These findings are consistent with a previous study which performed a similar epigenetic analysis with predicted causal AS SNPs.^96, 97^ In addition, AS-associated variants were shown to be enriched in regulatory elements, especially in enhancer regions, in related immune cells. Moreover, the AS upregulated genes show different enrichment bias in both gene and protein expression compared with AS downregulated genes. The AS upregulated genes are significantly enriched in monocytes, whereas AS downregulated genes showed enrichment in CD8^+^ T cell and NK cells in mRNA and protein expression.

The strong co-familiality between AS and IBD, the shared susceptibility loci, especially IL-23 pathway genes, suggest that gut microbial is involved in AS development. Differences in microbial signature in the terminal ileum of AS patients compared with healthy individuals have been demonstrated.^98^ Our finding that the genes specifically enriched in gut cells are also enriched in ‘response to bacterium’ and other similar Gene Ontologies further supports that AS status is influenced by the interaction among the host genetics, the intestinal microbiome and the immune response. Over-expression of the anti-microbial peptides alpha-defensin, phospholipase A2 and lysozyme by Paneth cells in human AS cases provides further support for the involvement of these cells and the gut mucosal immune system in AS pathogenesis.^99^ Bone cell types showed relative weak enrichment signals, consistent with the hypothesis that bone changes in AS are secondary to inflammatory processes rather than driven by bone-specific genetic effects. However, data on more AS-relevant tissues and cell types, such as enthesial and synovial cell populations, innate lymphoid cells and Paneth cells, is needed to identify more AS-relevant cell types and to further investigate the specific role of AS-associated variants in these cell types.

## Challenges

The two main remaining challenges in immunogenetic research in AS are to identify the variants responsible for the large proportion of heritability of the disease that remains unexplained, and determining the functional mechanisms underpinning those genetic associations. The sample size of GWAS that have been performed to date are relatively modest in comparison with the far larger studies that have been funded in other major immune-mediated diseases that have similar or lower disease prevalence, such as type 1 diabetes, IBD, and multiple sclerosis.^100^ Further large scale GWAS are clearly indicated.

In addition to discovering more genetic variants associations with AS, another challenge is fine mapping the casual variants through the associated signals. About 70% of the AS-associated SNPs are located in non-coding regions, making them difficult to be interpreted via traditional protein coding theory. Furthermore, these variants may function in specific cell types or tissues. Therefore, integrating cell-type specific multi-omics data, such as transcriptomic, epigenetic and proteomics, is necessary.

Besides genetic factors, only limited studies of epigenetic factors have been reported in AS.^95, 101^ Larger more systematic studies controlling for the profound effects of age, cigarette smoking and tissue type are required to properly investigate the role of epigenetic factors and AS, and also how genetic factors operate through epigenetic mechanisms to influence disease.

Whilst the main goal of genetic studies in AS is clearly to inform research ultimately leading to therapeutic or preventative approaches for the disease, given the high heritability of AS and its clinical manifestations, it is possible that genetic risk prediction may prove valuable in the disease. Genetic risk scores have been shown to have high discriminatory capacity between AS and healthy controls.^5^ Further development of these approaches using whole of GWAS data may prove even more informative given their ability to capture a higher proportion of disease heritability than analyses restricted to genome-wide significant loci.^102^ This may prove useful in identifying people either at high risk of disease to enable preventative approaches, or to distinguish that set of patients with suggestive early disease who truly have axial spondyloarthritis rather than non-inflammatory causes of back pain.^103^ Given the evidence that delay in implementing effective therapy is linked with worse outcome in AS,^104^ reducing the current substantial diagnostic delay in the disease would be a valuable contribution.

## Conclusions

GWAS is a hypothesis-free approach for testing the associations between hundreds of thousands of variants and phenotype. To date, at least 113 non-MHC variants have been identified as well as HLA-B*27 and these findings provide a wealth of new genetic information for AS, critical clues on biological pathways of development of AS and contributed to the development of new treatments. As mentioned above, at least several treatments targeting genes in IL-23R pathway, which was first uncovered in WTCCC GWAS, have been approved in AS or under clinical trials. In addition to genetic studies, functional studies of genetic variants in AS-relevant cell types will be necessary for understanding AS pathogenesis and developing novel treatments.

## Figures and Tables

**Table 1:** Major genome-wide association studies in AS

*Study*	*Year*	*Discovery cohort (case/control)*	*Coverage/variant types*	*Ethnicity*	*No. of non-MHC variants reaching genome-wide significance*
Burton *et al.* (WTCCC & TASC)^46^	2007	1000/1500	Non-synonymous variants only	European	2
Reveille *et al.* (TASC)^30^	2010	2053/5140	GWAS	European	4
Evans *et al.* (TASC)^47^	2011	3023/8779	GWAS	European	8
Lin *et al.*^49^	2011	1965/4301	GWAS	Han Chinese	3
Cortes *et al.* (IGAS)^20^	2013	10,619/15,145	Illumina Immunochip	European, East Asian and Latin American	24
Robinson *et al.*^48^	2016	5040/21,133	Illumina Exomechip	European	2
Ellinghaus *et al.*^19^	2016	8726/34,213	Illumina Immunochip	European and East Asian	113

Abbreviation: MHC, major histocompatibility complex.
